# Supplementary material for: Conditional chemoconnectomics (cCCTomics) as a strategy for efficient and conditional targeting of chemical transmission
Source: eLife. 2024 Apr 30;12:RP91927. doi: 10.7554/eLife.91927 (PMC11060718; doi:10.7554/eLife.91927)
Supplement: Figure 3—source data 3. [file elife-91927-fig3-data3.zip › Figure 3 source data3 part 1.zip!/Figure 3-Source data 3-part1/1063-13-CRISPResso_Report_8cz_fsdr.zip!/CRISPResso2Batch_report.html]

CRISPResso2 Report


# CRISPResso2

### Analysis of genome editing outcomes from deep sequencing data

##### CRISPResso Batch Output 8cz\_fsdr

1063\_13
1063\_14
1063\_15
1063\_16

##### Nucleotide percentages around guides

Composition of each base around the guide CGTCGAGCACATGACATCAA for the amplicon Reference

Data: Nucleotide frequencies

Data: Modification frequencies

##### Nucleotide percentages in the entire amplicon

Composition of each base for the amplicon Reference

Data: Nucleotide frequencies

Data: Modification frequencies

##### CRISPRessoBatch Insertions Across Samples for Reference

- Heatmap
- Line

Each row is a sample and each column is a position in the amplicon sequence. Each cell shows the percentage of insertions for the sample at that position relative to the amplicon. Guides for each sample are identified by a black rectangle.

Data: CRISPRessoBatch Modification Frequency Summary

Each line is a sample that indicates the percentage of insertions for the sample at that position relative to the amplicon. Guides are shown by a grey rectangle.

Data: CRISPRessoBatch Modification Frequency Summary

##### CRISPRessoBatch Deletions Across Samples for Reference

- Heatmap
- Line

Each row is a sample and each column is a position in the amplicon sequence. Each cell shows the percentage of deletions for the sample at that position relative to the amplicon. Guides for each sample are identified by a black rectangle.

Data: CRISPRessoBatch Modification Frequency Summary

Each line is a sample that indicates the percentage of deletions for the sample at that position relative to the amplicon. Guides are shown by a grey rectangle.

Data: CRISPRessoBatch Modification Frequency Summary

##### CRISPRessoBatch Substitutions Across Samples for Reference

- Heatmap
- Line

Each row is a sample and each column is a position in the amplicon sequence. Each cell shows the percentage of substitutions for the sample at that position relative to the amplicon. Guides for each sample are identified by a black rectangle.

Data: CRISPRessoBatch Modification Frequency Summary

Each line is a sample that indicates the percentage of substitutions for the sample at that position relative to the amplicon. Guides are shown by a grey rectangle.

Data: CRISPRessoBatch Modification Frequency Summary

If you like CRISPResso2 please support us by citing it in your work:   
Clement K, Rees H, Canver MC, Gehrke JM, Farouni R, Hsu JY, Cole MA, Liu DR, Joung JK, Bauer DE, Pinello L.  
CRISPResso2 provides accurate and rapid genome editing sequence analysis.   
Nat Biotechnol. 2019 Mar; 37(3):224-226. doi: 10.1038/s41587-019-0032-3. PubMed PMID: 30809026.

© Copyright Kendell Clement and Luca Pinello

Tweet about CRISPresso2!
